# Cytotoxicity of tranexamic acid to tendon and bone in vitro: Is there a safe dosage?

**DOI:** 10.1186/s13018-022-03167-5

**Published:** 2022-05-15

**Authors:** Scott M. Bolam, Arama O’Regan-Brown, Subhajit Konar, Karen E. Callon, Brendan Coleman, Nicola Dalbeth, A. Paul Monk, David S. Musson, Jillian Cornish, Jacob T. Munro

**Affiliations:** 1 Faculty of Medical and Heath Sciences, University of Auckland, Auckland, New Zealand; 2 Department of Orthopaedic Surgery, Auckland City Hospital, Auckland, New Zealand; 3 Department of Orthopaedic Surgery, Middlemore Hospital, Auckland, New Zealand; 4 Auckland Bioengineering Institute, University of Auckland, Auckland, New Zealand

**Keywords:** Arthroscopy, Intra-articular drugs, Tranexamic acid, Toxicity, Orthopaedics, Peri-articular tissues

## Abstract

**Introduction:**

Tranexamic acid (TXA) has been shown to be effective at reducing peri-operative blood loss and haemarthrosis in arthroplasty and arthroscopic soft tissue reconstructions. Intra-articular application, as an injection or peri-articular wash, is becoming increasingly common. Recent studies have shown TXA has the potential to be cytotoxic to cartilage, but its effects on human tendon and bone remain poorly understood. The aim of this study was to investigate whether TXA has any detrimental effects on tendon-derived cells and osteoblast-like cells and determine whether there is a safe dosage for clinical application.

**Materials and methods:**

Primary tendon-derived cells and osteoblast-like cells were harvested from hamstring tendons and trabecular bone explants, respectively, and analysed in vitro with a range of TXA concentrations (0 to 100 mg/ml) at time points: 3 and 24 h. The in vitro toxic effect of TXA was investigated using viability assays (alamarBlue), functional assays (collagen deposition), fluorescent microscopy and live/apoptosis/necrosis staining for cell death mechanisms in 2D monolayer and 3D collagen gel cell culture.

**Results:**

There was a significant (*P* < 0.05) decrease in tendon-derived cell and osteoblast-like cell numbers following treatment with TXA ≥ 50 mg/ml after 3 h and ≥ 20 mg/ml after 24 h. In tendon-derived cells, increasing concentrations > 35 mg/ml resulted in significantly (*P* < 0.05) reduced collagen deposition. Fluorescence imaging confirmed atypical cellular morphologies with increasing TXA concentrations and reduced cell numbers. The mechanism of cell death was demonstrated to be occurring through apoptosis.

**Conclusions:**

Topical TXA treatment demonstrated dose- and time-dependent cytotoxicity to tendon-derived cells and osteoblast-like cells with concentrations 20 mg/ml and above in isolated 2D and 3D in vitro culture. On the basis of these findings, concentrations of less than 20 mg/ml are expected to be safe. Orthopaedic surgeons should show caution when considering topical TXA treatments, particularly in soft tissue and un-cemented arthroplasty procedures.

**Supplementary Information:**

The online version contains supplementary material available at 10.1186/s13018-022-03167-5.

## Introduction

Intra-articular tranexamic acid (TXA; Trans-4-amino-methylcyclohexane-1-carboxylic acid) administration is effective in reducing blood transfusion rates in patients undergoing total hip and knee arthroplasty and is increasingly used in soft tissue reconstruction arthroscopic procedures [8, 10, 13, 29, 34, 42]. TXA acts as an antifibrinolytic agent, which competitively inhibits the lysine-binding sites in plasmin and plasminogen activator molecules, to inhibit the breakdown of fibrin clot [15, 24, 39]. TXA can be administered via intravenous (IV), oral, intra-articular and peri-articular routes.

There remains no consensus on the optimal route or dose to administer TXA for most orthopaedic procedures. Previous studies performed in vitro have demonstrated that TXA at 10 ug/ml results in 80% coagulation. This concentration can be achieved with an IV dose of 10 mg/kg that leads to concentrations of < 20 ug/ml 1 h after administration [21, 28]. Several clinical studies have found reduced blood loss with relatively low concentrations (10–20 mg/ml) of intra-articularly administered TXA, implying that concentration up to 20 mg/ml can be effective and non-harmful [1, 6, 17, 33, 35, 38, 42].

IV administration of TXA is a widely used peri-operative haemostatic treatment and is generally considered safe [32]. However, some studies have associated it with acute renal impairment, increased thromboembolic events and post-operative seizures [14, 15, 25]. Topical administration could avoid these risks by directly delivering TXA to the target site at significantly lower systemic exposure than IV TXA. Intra-articular TXA may also more effectively reduce intra-articular bleeding during arthroscopic procedures to improve visualisation and reduce post-operative morbidity from haemarthrosis [4, 18, 20, 33].

Previous in vitro studies have suggested that TXA concentrations less than 20 mg/ml do not significantly reduce cell viability in chondrocytes, tenocytes, synoviocytes and periosteum-derived cells; however, there is not yet a clear consensus and the evidence is sometimes conflicting [5–7, 23]. The majority of previous studies have focussed on toxicity to cartilage [3, 16, 23, 31, 36, 37]. Importantly, only two previous studies have considered toxic effects of TXA on tendon [7, 23] and only one previous study has considered its effects on bone in vitro [5]. The toxic effects of TXA on tendon are particularly important to consider as adult tendon has poor regenerative capacity, and therefore, any additional insult could significantly impair surgical outcomes, particularly after rotator cuff surgery or anterior cruciate ligament reconstructions with tendon grafts. Bone effects are also important, as bone regeneration is important for osseointegration of implants after arthroplasty and spinal fusion surgery, and thus, negative effects may influence the risk of aseptic loosening or non-union.

In this study, our hypothesis was that TXA causes dose- and time-dependent toxicity to human tendon and bone. We aimed to investigate the toxic effects of TXA on human tendon-derived cells and osteoblast-like cells and to use the information obtained to suggest a safe dosage for intra-articular TXA administration around tendon and bone. This is the first study to investigate the toxicity of TXA on human tendon-derived cells and osteoblast-like cells using 2D and 3D cell culture models.

## Methods

### Ethical approval

Human bone and tendon sample collection at the time of orthopaedic surgery was approved by the New Zealand Ministry of Health Northern Regional Ethics Committee (NTX/05/06/058/AM14), and all participants provided written informed consent prior to collection of samples. Individual donor tissues were collected from the excess hamstring tendon autograft of three healthy patients undergoing anterior cruciate ligament reconstruction aged 25 (male) and 41 and 66 (both females). Trabecular bone explants were harvested from three patients undergoing total knee arthroplasty aged 65 (male) and 71 and 80 (both females) (Additional File 1: Table 1). Patients were excluded from the study if they were: < 18 years; had previous knee surgery; or had other knee pathology, rheumatoid arthritis or systemic inflammatory disease.

### Reagents

Dulbecco’s modified Eagle’s Medium (DMEM), DMEM/Nutrient Mixture F-12 (DMEM/ F-12), penicillin–streptomycin mixture (10,000 U/mL), and foetal bovine serum (FBS) were obtained from Gibco (Thermo Fisher Scientific, Waltham, MA, USA). Bovine serum albumin (BSA) was obtained from Immuno-Chemical Products Ltd. (Auckland, New Zealand). TXA (Trans-4-amino-methylcyclohexane-1-carboxylic acid) was obtained from Apollo Scientific (Stockport, UK). The cells in each experiment were exposed to TXA in ranges of concentrations to mimic the range that could be expected following intra-articular administration (0, 10, 20, 35, 50 or 100 mg/mL), with specific graduations of TXA chosen to better define the toxic threshold previously predicted to be 20 mg/ml [4, 6, 18, 20, 33].

### Primary tendon-derived cells 2D cell culture

Primary tendon-derived cell culture was performed as previously described [9]. Briefly, excess tissue from healthy human hamstring grafts was harvested and kept hydrated at 4 °C until use. Tendon tissue was roughly cut into pieces smaller than 0.5 cm^2^. The tissue was then digested in 0.5 mg/mL of dispase (Sigma-Aldrich) and 0.5 mg/mL of collagenase (Sigma-Aldrich) in DMEM/F-12 with 10% FBS at 37 °C until all extracellular matrix (ECM) had been digested. The cell suspension was then passed through a cell strainer, washed and re-suspended in enzyme-free media. Cells were cultured in DMEM/F-12 with 10% FBS in 75-cm^2^ flasks (Corning, Corning, NY, USA) and incubated at 37 °C with 5% carbon dioxide until confluent. Cell cultures were frozen down for a period before being used for these experiments.

### Primary osteoblast-like cells 2D cell culture

Primary human osteoblast-like cells were grown from trabecular bone explants obtained from patients undergoing knee arthroplasty, as previously described [26]. In brief, trabecular bone explants were chopped into small bone chips and the bone marrow removed by repeated washes. The bone chips were placed in in 75 cm^2^ flasks with DMEM containing 10% FBS and 5 μg/ml L-ascorbic acid 2-phosphate (AA2P) and incubated at 37 °C with 5% carbon dioxide. When osteoblast outgrowth was first observed, the medium was refreshed and the outgrowing osteoblasts, having twice weekly media changes, were grown until confluent.

### 3D cell culture in tendon-derived cells and osteoblast-like cells

For 3D cultures, 3 mg/mL rat collagen type I gels were used (BD Biosciences), as previously described [22, 27]. Once isolated as described above, primary human tendon-derived cells and osteoblast-like cells suspended in culture medium were seeded in 50 μL collagen gels at 10^4^ cells per gel (2 × 10^5^ cells ml^−1^). For 3D cultures of primary osteoblast-like cells, hydroxyapatite particles were incorporated into the collagen gels at a concentration of 25 mg/mL, as previously described [26]. Gels were allowed to set at 37 °C for 1 h before addition of DMEM + 10% FBS + 10 μg/mL AA2P culture medium.

### Cell viability assays

Primary human tendon-derived cells and osteoblasts were seeded in 24-well plates (Greiner Bio-One, Kremsmünster, Austria), at a density of 2.5 × 10^4^ cells/well, and cultured in either DMEM/F-12 with 5% FBS (tendon-derived cells) or DMEM with 5% FBS (osteoblast-like cells). After 24 h, cultures were growth-arrested for a further 24 h in either DMEM/F-12 with 1% FBS (tendon-derived cells) or DMEM with 0.1% bovine serum albumin (BSA) and 5 μg/mL AA2P (osteoblast-like cells). Fresh growth arresting media and the TXA solution (0, 10, 20, 35, 50 or 100 mg/mL) were added for either 3 or 24 h. After the 3 h designated time points, media was exchange for fresh growth arresting media for a further 21 h. Cell growth was measured by adding alamarBlue® (Life Technologies, ThermoFisher Scientific Inc.) at 5% of final concentration in well for 4 h at 37 °C. Following this incubation, 200 μl of the alamarBlue conditioned medium was transferred to a 96-well plate (Greiner Bio-One) and fluorescence (excitation 540 nm; emission 630 nm) was read using a Synergy 2 multi-detection microplate reader (BioTek Instruments Inc., Winooski, VT, USA). The change in alamarBlue is expressed here as a ratio of the untreated control fluorescence readings.

In the 3D collagen gels, primary human tendon-derived cells and osteoblast-like cells were seeded as described above. Media changes and TXA solution were added and removed at the same time points, with the exception that after a fresh media change at 24 h from the addition of TXA solution, alamarBlue was added to media for 24 h before a reading was taken.

### Tendon-derived cells collagen deposition

Tendon-derived cells were seeded in 24-well plates, at a density of 7.5 × 10^4^ cells/well, and cultured in DMEM/F-12 with 5% FBS for 24 h, as previously described [9]. Cells were then incubated in fresh DMEM F-12 1% FBS and 50 µg/ml AA2P with the TXA solution (0, 10, 20, 35, 50 or 100 mg/mL). At the end of the 24 h treatment period, cells were then incubated in fresh media for 72 h. Then, cells were fixed with Bouin’s solution (71% saturated picric acid, 24% formalin, 5% 0.5 M acetic acid) for 30 min and stained with 0.1% Sirius red dissolved in saturated picric acid for 1 h. At the end of this incubation, cells were washed with 0.01 M hydrochloric acid five times and left to air dry. The dye was released using 0.1 M sodium hydroxide, and 200 μl of the released dye was transferred to a 96-well plate. Absorbance was measured at 570 nm using a Synergy 2 multi-detection microplate reader. There were 4 wells in each TXA concentration group.

### Live cell staining in 2D cell culture model

Cells were cultured and treated as described above in 2D models. At the conclusion of the culture period, cells were washed in PBS. Cells were then stained for 10 min at 37 °C with 2 μM calcein-AM (Invitrogen, Carlsbad, CA, USA) in PBS, washed in fresh PBS and viewed immediately using fluorescent microscopy. Calcein-AM is converted to a green fluorescent product within live cells. Cells were then imaged using fluorescent microscopy using a Zeiss LSM 710 Inverted Confocal Microscope (Carl Zeiss, Oberkochen, Germany).

### Apoptotic, necrotic and healthy cell staining in 3D culture model

To determine the mode of cell death, cells were cultured and treated as described above in 3D culture models. Following either 3 or 24 h of TXA exposure, cells were washed twice with PBS, then stained with Apoptotic, Necrotic & Healthy Cells Quantitation Kit (Biotium, Hayward, CA, USA) as per the manufacturer’s instructions. Cells were incubated with the staining solution for 15 min at room temperature, washed twice with binding buffer and then imaged using fluorescent microscopy using a Zeiss LSM 710 Inverted Confocal Microscope.

### Statistical analysis

Tendon-derived cells and osteoblast-like cells from the same donor were test in parallel with exposure to different TXA concentrations and duration. All results are shown as combined data from three donors with mean ± standard error of the mean (SEM). All statistical analysis was performed either using one-way or two-way analysis of variance (ANOVA) test with Dunnett’s post hoc analysis using the GraphPad Prism 8 software (GraphPad Software, San Diego, California). A *P* value of < 0.05 was considered statistically significant.

## Results

### Tendon-derived cells

#### 2D model

**Fig. 1:**
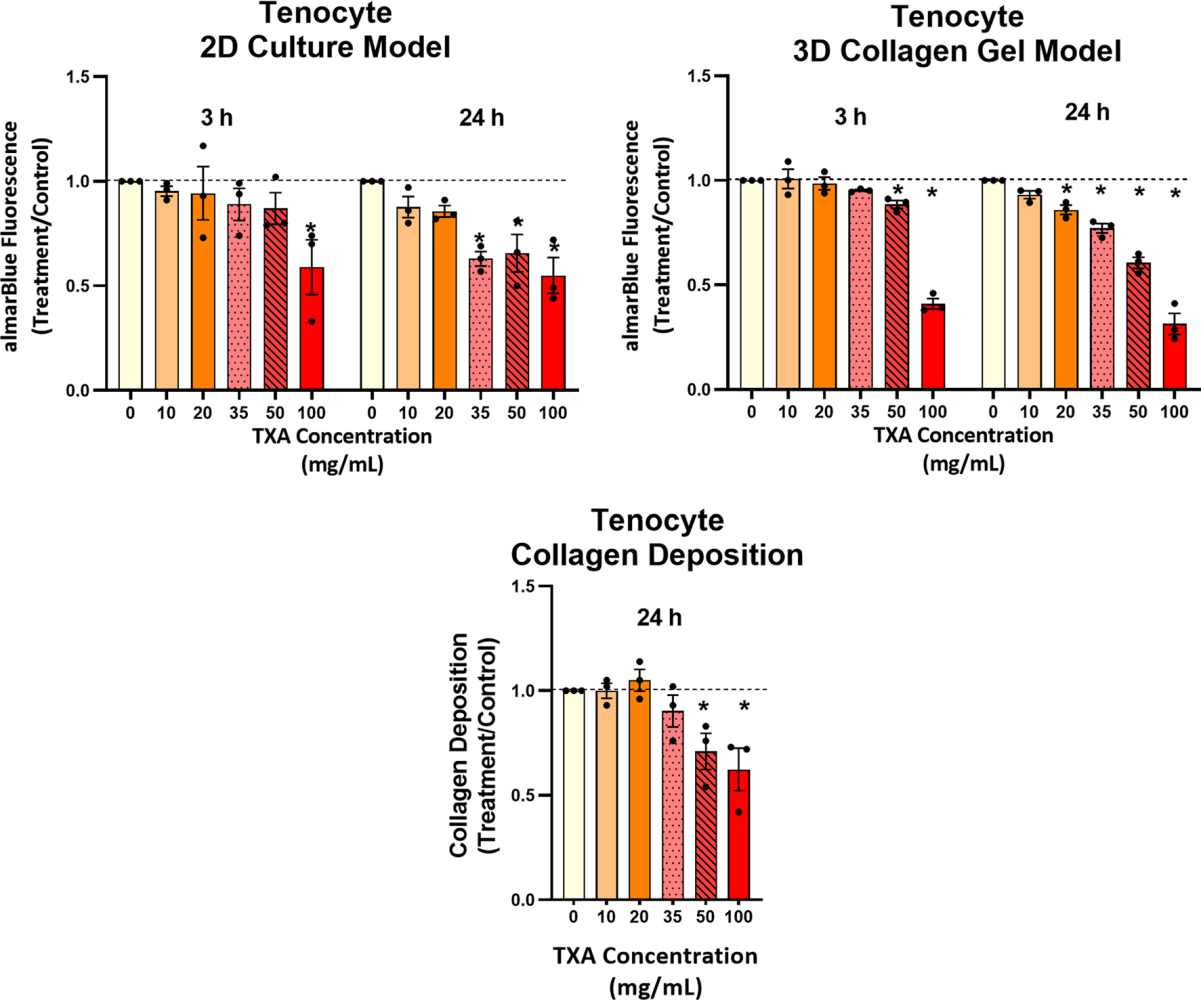
Graphs showing the cell viability of tendon-derived cells in 2D (**A**) and 3D collagen gel (**B**) culture after 3 and 24 h of TXA exposure ranging from 0 to 100 mg/mL. Collagen production in tendon-derived cells after 24 h of TXA exposure is shown in (**C**). *P < 0.05 compared to control. Data are presented as combined data from three donors with mean ± SEM

Tendon-derived cell viability was decreased 41% after 3 h exposure with TXA 100 mg/mL (*P* = 0.0027). After 24 h, TXA 35 mg/mL induced a significant 37% decrease in tendon-derived cell viability (*P* = 0.0070). Additionally, concentrations of TXA 50 and 100 mg/mL also caused a significant reduction in cell numbers in both (34% (P = 0.0129) and 45% (*P* = 0.0011), respectively) (Fig. 1).

**Fig. 2:**
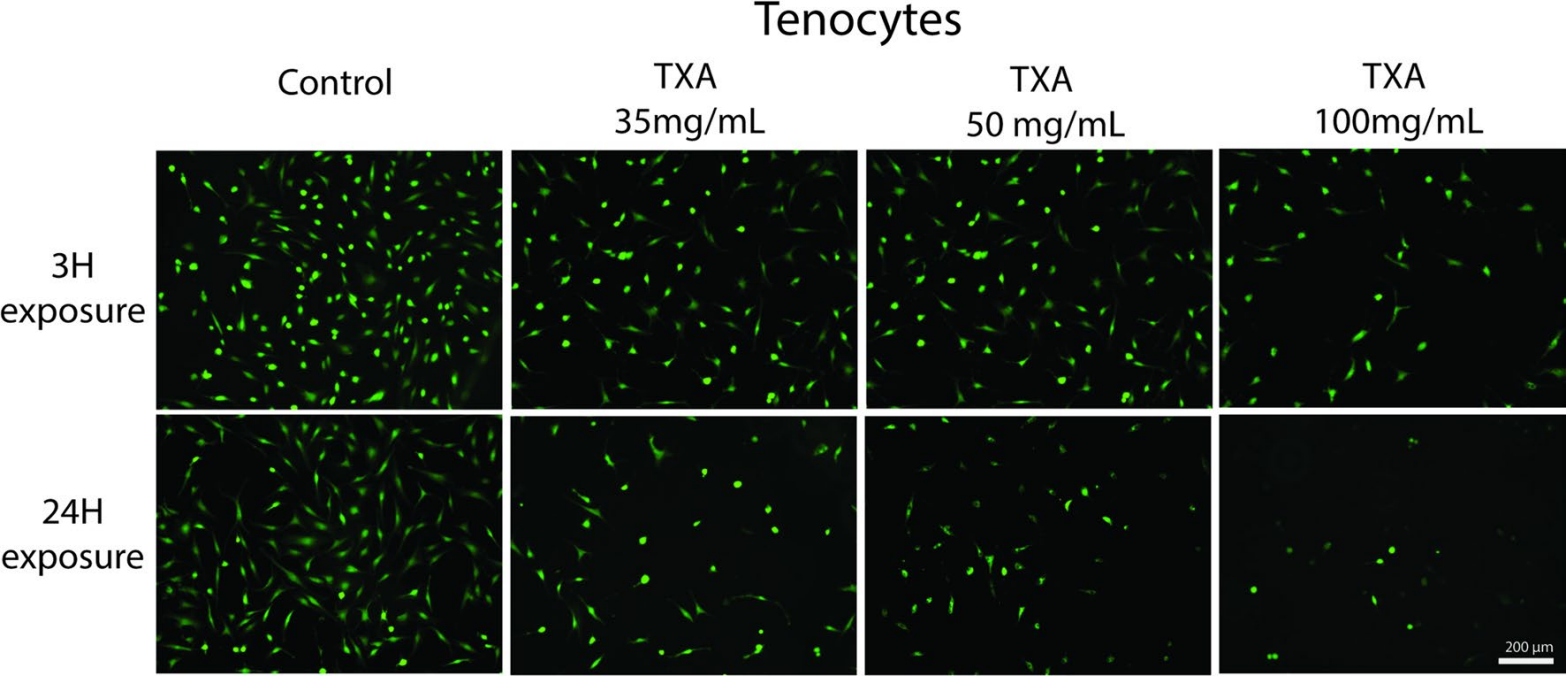
Fluorescence imaging showed fewer cells and atypical morphology (from a spindle-like phenotype to a polygonal shape) in tendon-derived cells at TXA100 mg/mL with 3 h exposure and at 35 mg/mL and above with 24 h exposure

Qualitative analysis using fluorescent staining demonstrated a similar pattern of reduced cell numbers with increasing concentrations of TXA, consistent with alamarBlue results. Additionally, the morphology of the tendon-derived cells changed from spindle-like in the control cells, to a polygonal shape, at TXA 100 mg/mL after 3 h and TXA 35 mg/mL and above after 24 h (Fig. 2).

Concentrations of TXA 50 and 100 mg/mL after 24 h exposure resulted in significantly reduced collagen deposition, as quantified by Sirius red dye release. TXA 50 mg/mL and 100 mg/mL reduced collagen deposition by 29% (*P* = 0.0411) and 38% (*P* = 0.0083), respectively.

#### 3D collagen gel model

Similar to 2D models, increasing the concentration of TXA around tendon-derived cells seeded in collagen gels resulted in dose-dependent reductions in cell viability (Fig. 1). After 3 h of exposure, cell viability significantly decreased by 27.2% (*P* = 0.0252) and 38.0% (*P* < 0.0001) with TXA 50 mg/mL and TXA 100 mg/mL in tendon-derived cells, respectively. At 24 h, cell viability was reduced significantly by 36.2% (*P* = 0.005) with TXA 20 mg/mL and continued in a dose-dependent manner. The greatest decrease in viability was seen at TXA 100 mg/mL, with cell viability decreasing 71.4% (*P* < 0.0001). There was no effect on tendon-derived cell viability with TXA concentrations below 20 mg/mL.

### Osteoblast-like cells

#### 2D model

**Fig. 3:**
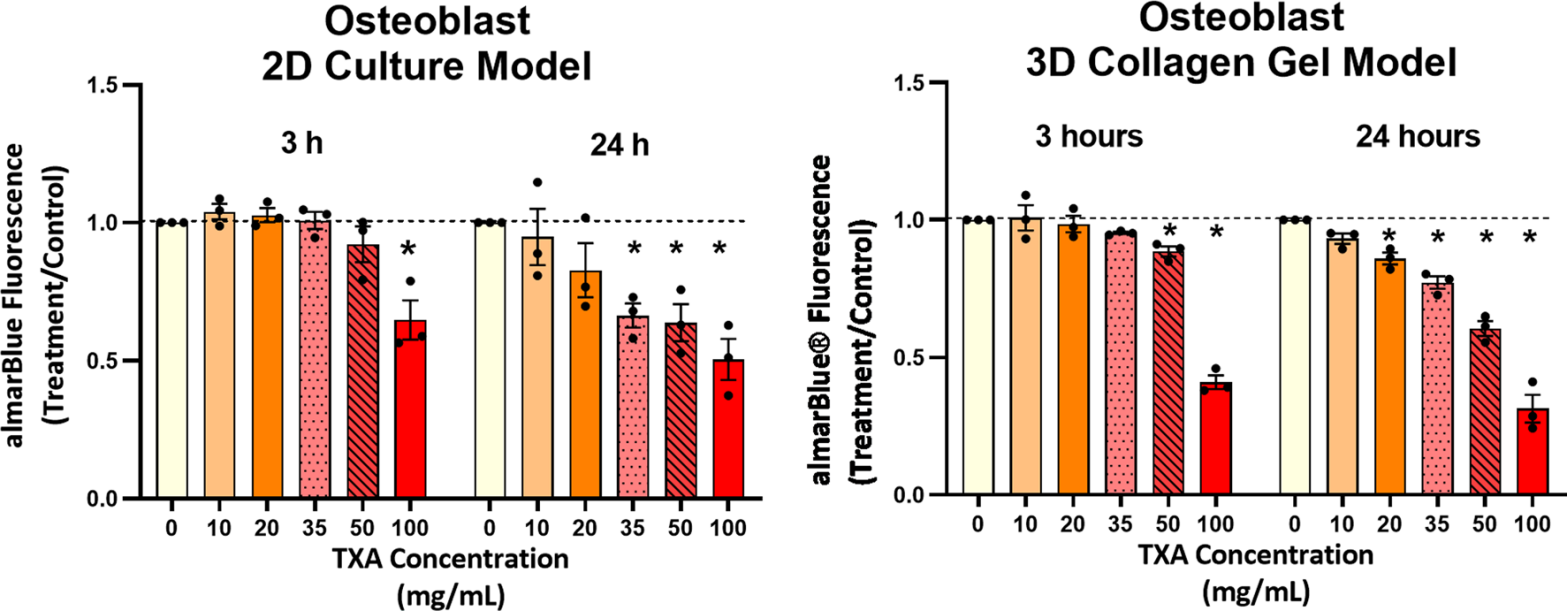
Graphs showing the cell viability of osteoblast-like cells in 2D (**A**) and 3D collagen/hydroxyapatite gel (**B**) culture after 3 and 24 h of TXA exposure ranging from 0 to 100 mg/mL. Data are presented as combined data from three donors with mean ± SEM

As with tendon-derived cells, osteoblast-like cell viability was decreased 42% after 3 h exposure with TXA100 mg/mL (*P* = 0.0002). After 24 h exposure, TXA 35 mg/mL led to a significant fall in cell vitality by 38% (*P* = 0.0002). Increasing concentrations of TXA 50 and 100 mg/mL also caused a significant reduction in cell numbers in osteoblasts (40%, (*P* < 0.0001) and 58%, (*P* < 0.0001), respectively) (Fig. 3).

**Fig. 4:**
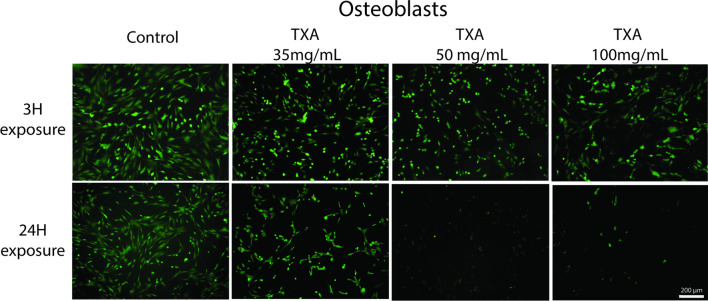
In osteoblast-like cells, fluorescence imaging showed fewer cells and atypical morphology (more narrowed and rounded) at TXA100 mg/mL with 3 h exposure and at 35 mg/mL and above with 24 h exposure

Findings that were consistent with alamarBlue results were observed with fluorescent microscopy, with reduced cells number at TXA 100 mg/mL after 3 h and TXA 35 mg/mL and above after 24 h. Osteoblast-like cell was also noted to change from their healthy spindle-shaped appearance to become more narrowed and rounded at TXA concentrations that also lead to reduced viability (Fig. 4).

#### 3D collagen/hydroxyapatite gel model

As with 2D models, increasing the concentration of TXA around collagen/hydroxyapatite gels resulted in dose-dependent reductions in cell viability. After 3 h exposure to TXA 35 mg/mL, TXA 50 mg/mL and TXA 100 mg/mL, osteoblast-like cell viability fell by 13% (*P* = 0.0148), 30% (*P* < 0.0001) and 56% (*P* < 0.0001), respectively. At 24 h significant reductions in cell viability were seen from TXA 20 mg/mL (29% reduction, *P* < 0.0001) and continued in a dose-dependent manner. The greatest reduction in viability was seen at TXA 100 mg/mL, with a 62% (*P* < 0.0001) decrease (Fig. 3).

### Mode of cell death

**Fig. 5:**
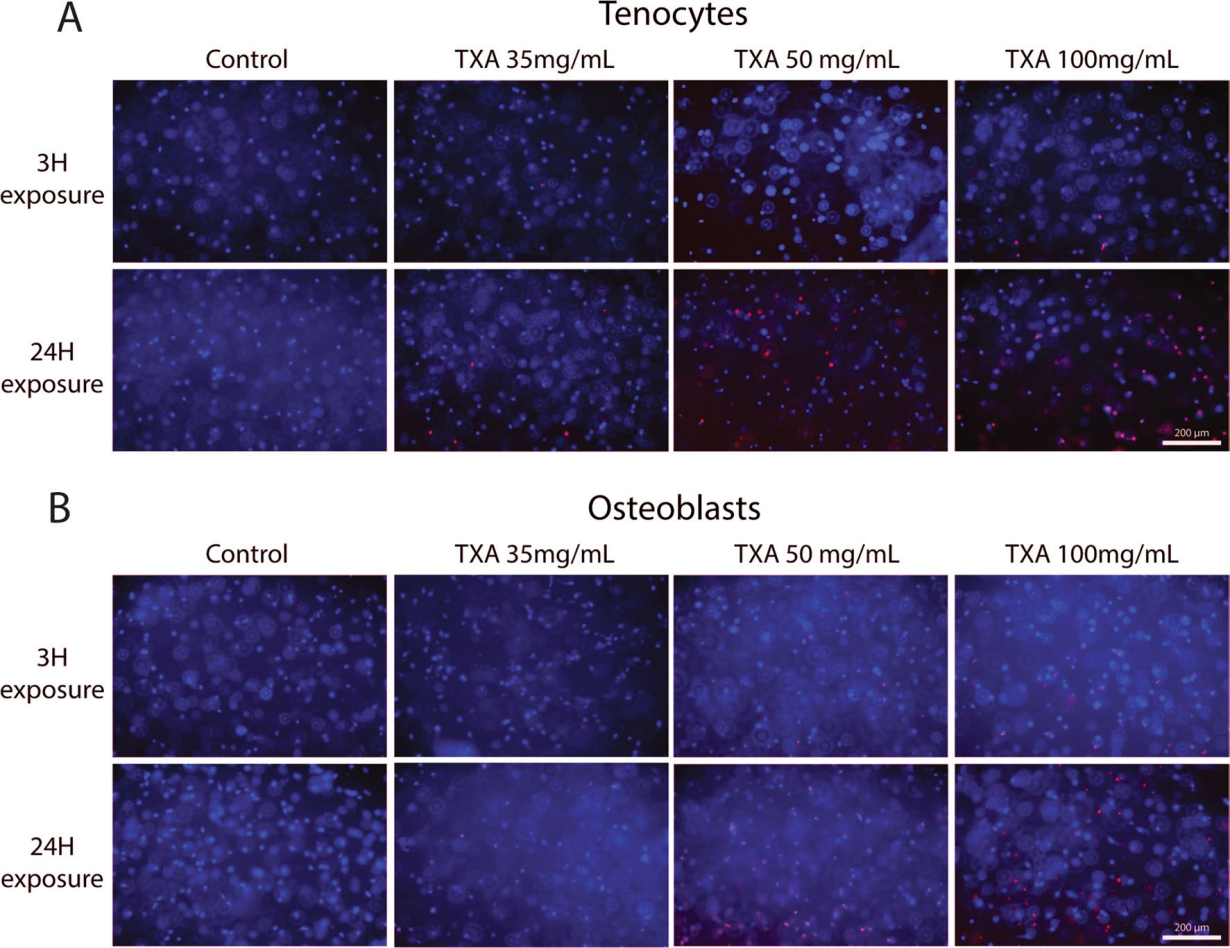
Apoptotic (red), necrotic (green) and healthy (blue) cell staining in tendon-derived cells (**A**) and osteoblast-like cells (**B**) in 3D gels demonstrated apoptosis (red stained cells) to be the main mechanism of cell death at TXA 100 mg/mL with 3 h exposure and at 35 mg/mL and above with 24 h exposure, with a dose-dependent response

In order to assess mode of cell death for each cell type, further qualitative analysis using apoptotic, necrotic and healthy cells staining was performed in 3D cell culture models. Treatments with TXA 100 mg/mL for 3 h and TXA 35 mg/mL and above for 24 h caused an increase in Annexin V binding (red, indicating activation of apoptosis) in both tendon-derived cells and osteoblast-like cells. However, no evidence of ethidium homodimer III binding (green, indicating cell death via necrosis) was observed at any concentration or time point of TXA exposure. This suggests TXA-mediated cell death is orchestrated via an apoptotic cellular process in both cell types (Fig. 5).

## Discussion

The main finding of this in vitro study is that TXA causes clear dose-dependent toxicity to human tendon-derived cells and osteoblast-like cells. After 3 h, TXA 50 mg/mL and above caused significant decreases in cell viability in 2D and 3D cell culture models. TXA-induced cell death was observed to occur through an apoptotic cellular mechanism. Importantly, concentrations of TXA less than 20 mg/mL did not cause significant toxicity, even with prolonged 24 h exposure. This suggests that TXA can be administered safely below this toxic threshold around healthy tendon and bone. This has important clinical implications as intra-articular administration of TXA for arthroplasty and soft tissue reconstructions surgeries where TXA has been shown to reduce blood loss, inflammation and post-operative swelling, benefitting patients with a more rapid recovery [8, 10, 13, 29, 34, 42].

There is no consensus on the optimal dose or route of TXA for most orthopaedic procedures. Doses of TXA for the intra-articular administration range from 250 mg to 3 g, which correspond to concentrations of TXA between 15 and 100 mg/mL [10, 21, 37, 41]. Surgical practices including prolonged surgical soaking, intra-articular infusion and peri-articular tissue injection into an enclosed joint space should be undertaken with caution, given that high concentrations were shown to lead to increased cell death in bone and tendon. Previous in vitro studies have demonstrated that a concentration of 10 μg/ml can produce 80% coagulation, which can be achieved with an IV dose of 10 mg/kg [21, 28]. Several recent studies have reported that relatively low concentrations (10–20 mg/ml) of topically administered TXA, below the threshold for toxicity found in this study, can effectively reduce blood loss after arthroplasty procedures [21, 28].

There is a lack of understanding about how rapidly TXA diffuses out of the joint space following intra-articular administration or to what degree it is diluted by bleeding or synovial fluid. In addition, the dynamic environment of the joint space and the presence of other cells types (inflammatory cells and platelets) could have a role in the absorbance of TXA into surrounding tissues [6]. During arthroplasty, TXA has been shown to diffuse rapidly into synovial fluid after IV administration, equal to serum concentrations, and the half-life in synovial fluid is 3 h [2]. Over 90% of TXA would be expected to be cleared after 12 h or four half-lives. The 3-h exposure time in this study therefore appears to be the most clinically relevant. The 24-h exposure time was included to determine whether a threshold value for toxicity existed with prolonged TXA exposure.

Our findings in tendon-derived cells are consistent with previous in vitro studies which demonstrated TXA concentrations of 50 mg/ml and above to be cytotoxic in monolayer culture after 4 h [7, 23]. In osteoblast-like cells, TXA concentrations of 35 mg/ml and above were shown to be toxic to osteoblast-like cells after 3 h and there were significantly reduced cells numbers with TXA 20 mg/mL and above after 24 h. Only one previous in vitro study has considered the direct effects of TXA in osteoblast derived from mice at relatively low concentrations (up to 1 mg/ mL) [5]. Interestingly, they reported increased cell proliferation and matrix mineralisation in bone marrow-derived osteoblast that was associated with increased expression of genes involved in osteoblast differentiation and ECM synthesis with long-term exposure over ten days. Cuellar et al. [11] found that TXA administered 1000 mg/kg via an intra-peritoneal injection at the time of in vivo spinal fusion surgery in a mouse study did not affect fusion bone volume. However, it is unclear what local concentration of TXA would have been achieved at the spinal fusion site. Other studies have investigated the effects of TXA using osteogenically differentiated human mesenchymal stromal cells (hMSCs) and human periosteum-derived cells (hPDCs) [12, 40], instead of osteoblasts. Demol et al. [12] exposed hPDCs to a relatively lower concentration of TXA of 0.5 mg/ml for up to 21 days and found that no effects on cell proliferation or calcium mineralisation were observed. Wagenbrenner et al. [40] found that hMSCs treated with TXA 50 mg/mL for 24 h had decreased cellular proliferation rates, which is in agreement with our findings.

In this study, the toxic effect of TXA on tendon-derived cells and osteoblasts was evident in both the 2D and 3D models. In 2D studies, decreased viability was observed at 100 mg/mL after 3 h in both cell types, whereas in the 3D collagen gel study decreased viability was seen at 50 mg/mL and 35 mg/mL in tendon-derived cells and osteoblast-like cells, respectively. 3D collagen gels not only provide a better representation of the in vivo environment, but have also been shown to lower basal rates of proliferation in tenocytes and osteoblasts compared to monolayer cell culture [19, 22]. This difference might have contributed to the increased sensitivity of both cell types in 3D-model to TXA. In a similar study using chondrocytes, Parker et al. [30] found lower susceptibility to TXA in 3D gelatin-methacryloyl hydrogels compared to monolayer culture, which is possibly explained by the composition of this hydrogel having a greater protective barrier effect to TXA. In a previous study, tendon explants treated with 100 mg/ml TXA for 16 h were found to have a significant increase in cell death, as determined by imaging the explant surface using confocal microscopy with live/dead staining [23]. This is possibly because the weak negative charge of the TXA inhibits its penetration through the ECM, thus providing a protective effect. The ECM may also be acting as buffer, effectively lowering the concentrations of TXA interacting with the cells.

There are clear limitations with this in vitro study. Firstly, it does not account for the TXA being diluted with synovial fluid and cleared from the joint that occurs in vivo. TXA has been shown to have a half-life of 3 h in the synovial fluid of a closed joint following IV administration; therefore, we believe our in vitro findings are clinically relevant. We used a validated 3D culture model with collagen (and hydroxyapatite for bone only) to approximate the bone and tendon environment; however, this is not a perfect representation of the tendon or bone ECM. We also did not investigate the mechanism of action through which TXA is inducing cell death, and it is possible that TXA is having effects other than cell death on intra-articular tissues. Future studies should investigate whether different concentrations of TXA affect gene and protein expression of tendon- and bone-specific markers. Finally, we recognise that these in vitro findings may not necessarily translate into adverse clinical outcomes in patients. There is need for further human clinical trials to evaluate the early and late outcomes in patients treated with TXA before making definitive clinical recommendations.

Overall, TXA treatment demonstrated dose- and time-dependent cytotoxicity to human tendon-derived cells and osteoblasts-like cells with concentrations 20 mg/ml and above in isolated 2D and 3D in vitro culture. Based on the findings of this study, orthopaedic surgeons should administer intra-articular TXA treatments with caution around tendon and bone, particularly in soft tissue and un-cemented arthroplasty procedures. Importantly, TXA appeared to be able to be administered below a toxic threshold of less than 20 mg/mL without causing any reduction in cell viability, even with prolonged exposure.

## Supplementary Information

**Additional file 1.** Individual patient demographics, biomorphic measurements and concurrent conditions.

## Data Availability

All data generated or analysed during this study are included in this published article.

## Abbreviations

AA2P: L-ascorbic acid 2-phosphate
ANOVA: Analysis of variance
DMEM: Dulbecco’s modified Eagle’s medium
DMEM/F-12: DMEM/Nutrient Mixture F-12
ECM: Extracellular matrix
FBS: Foetal bovine serum
hMSCs: Human mesenchymal stromal cells
hPDCs: Human periosteum-derived cells
IV: Intravenous
SEM: Standard error of mean
TXA: Tranexamic acid
2D: Two-dimensional
3D: Three-dimensional
