# Supplementary material for: Cytotoxicity of tranexamic acid to tendon and bone in vitro: Is there a safe dosage?
Source: J Orthop Surg Res. 2022 May 15;17:273. doi: 10.1186/s13018-022-03167-5 (PMC9107642; doi:10.1186/s13018-022-03167-5)
Supplement: Supplementary file 1 — Additional file 1. Individual patient demographics, biomorphic measurements and concurrent conditions. [file 13018_2022_3167_MOESM1_ESM.docx]

Additional file 1. Individual patient demographics, biomorphic measurements and concurrent conditions

| **Tissue Type** | **Indication for surgery** | **Surgery type** | **Gender** | **Age** | **BMI (kg/m^2^)** | **Concurrent conditions** |
| --- | --- | --- | --- | --- | --- | --- |
| Femoral condyle trabecular bone explants | Knee osteoarthritis | Total knee replacement | Female | 71 | 35.1 | Hypothyroidism, hypertension |
| Femoral condyle trabecular bone explants | Knee osteoarthritis | Total knee replacement | Male | 65 | 34.6 | Non-insulin dependent type II diabetes |
| Femoral condyle trabecular bone explants | Knee osteoarthritis | Total knee replacement | Female | 80 | 36.5 | Hypertension |
| Hamstring tendon autograft | Anterior cruciate ligament rupture | Anterior cruciate ligament reconstruction | Male | 25 | 34.4 | - |
| Hamstring tendon autograft | Anterior cruciate ligament rupture | Anterior cruciate ligament reconstruction | Female | 66 | 20.8 | - |
| Hamstring tendon autograft | Anterior cruciate ligament rupture | Anterior cruciate ligament reconstruction | Female | 41 | 28.5 | - |
